# Has China’s New Round of Collective Forest Reforms Reduced Forest Fragmentation? A Case Study of the Beijing–Tianjin–Hebei Region

**DOI:** 10.3390/ijerph19106183

**Published:** 2022-05-19

**Authors:** Shuning Zhu, Jinlong Liu, Hao Xu, Lingchao Li, Wentao Yang

**Affiliations:** 1 School of Agricultural Economics and Rural Development, Renmin University of China, Beijing 100872, China; zhushuning@ruc.edu.cn (S.Z.); liujinlong@ruc.edu.cn (J.L.); xhao1110@ruc.edu.cn (H.X.); 2 School of Economics & Management, Beijing Forestry University, Beijing 100083, China; 3 School of Soil & Water Conservation, Beijing Forestry University, Beijing 100083, China

**Keywords:** reform of collective forestland tenure, forest fragmentation, landscape indices, fixed-effect model

## Abstract

The new wave of reform of collective forestland tenure (NRCFT) in China is considered an important policy for achieving sustainable management of forest resources. The purpose of this study is to investigate the influence of NRCFT on forest fragmentation in the Beijing–Tianjin–Hebei region of China based on a fixed-effects model. The forest fragmentation was analyzed based on the remote sensing images of Landsat and landscape pattern indices in the Beijing–Tianjin–Hebei region from 2000 to 2018. The results showed that (1) The NRCFT has significantly contributed to reducing forest fragmentation. (2) The effect of economic growth on forest fragmentation showed an inverted U-shape. (3) The implementation of the Grain for Green Program (GGP) and the transformation of rural energy consumption significantly reduce the degree of forest fragmentation. This study has crucial implications for formulating policies, achieving good forest governance, and reducing forest fragmentation.

## 1. Introduction

Forests are one of the important terrestrial ecosystems and play a decisive role in global biodiversity conservation. Socio-economic development [1,2,3,4,5] and rapid population growth have resulted in environmental pollution and expansion of land demand [6,7,8,9,10,11]. Global forests are facing the threat of area reduction and quality degradation. Forest fragmentation refers to the process by which large, continuous forests are fragmented into smaller, independent patches as a result of deforestation and land-use change [12,13]. As an important symptom of forest degradation, it causes a series of negative environmental effects such as reduced habitat area for animals [14,15,16,17,18], energy imbalance, and more inefficient material flow within the forest ecology [19,20,21,22,23].

To curb forest degradation, forest conservation practices based on decentralization reforms are gradually taking place in developing countries. Decentralization improves community livelihoods and achieves sustainable forest development [24,25,26,27] by harnessing indigenous knowledge, increasing accountability, and legitimizing forest tenure [28,29,30]. China has undergone a series of fundamental changes in forest tenure arrangements since 1949 [31,32,33]. The land reform movement in the early 1950s returned forestland to individual farmers through equalization of hills and forests, and farmers enjoyed full forest rights. Forming the private forest tenure system in the early years of China’s founding [34]. In the late 1950s, private forestry went through four stages: mutual aid groups, primary cooperatives, senior cooperatives, and people’s communes. Private rights to forestland and trees were gradually collectivized. Since 1980, influenced by the family contract responsibility system in China’s agricultural sector, China promulgated the “Three Fixes policy” for forestry, and nearly 70% of collective forests were allocated to households [35]. With the liberalization of the timber market in 1985, indiscriminate logging occurred in the southern collective forest areas [36,37,38]. The central government had to abolish the policy in 1987. In the early 1990s, with the reform of China’s market economy system, forestry began to introduce market mechanisms, and the trend of multiple property rights models sprouted in collective forest property rights. Until the implementation of the new wave of reform of collective forestland tenure (NRCFT) in 2003, the security of individual household tenures has been improved and forests have been effectively protected.

A reasonable forest property rights system has an important role in the conservation of forest resources [39,40,41,42]. Most studies on the effects of NRCFT have focused on three aspects: social effects, economic effects, and ecological effects.

- In terms of social effects, most studies have concluded that NRCFT enhances the stability of property rights in forestland [43,44,45] and reduced the occurrence of forest rights disputes [46,47,48]. NRCFT has also promoted the transfer of forest rights and given rise to forestry cooperative organizations [49,50,51], which stabilized the basic rural management system [52].
- In terms of economic effects, several scholars have affirmed the income-generating effects of NRCFT on rural households from the perspective of property rights theory [53]. Some research confirmed that NRCFT has increased the nonfarm employment and income of rural households by optimizing the allocation of rural labor [54,55]. However, from the perspective of economies of scale, NRCFT led to higher land tenure fragmentation, which reduces the scale effect of forestry operations and thus affects forestry output [56,57,58].
- At the level of ecological effects of NRCFT, effective property rights arrangements are considered one of the prerequisites for forest restoration [59,60]. Clear property rights can “internalize” the externalities of public goods and avoid the phenomenon of “tragedy of the commons” [61,62]. NRCFT provides farmers with more integrated and secure forestland rights [63,64], which reduced deforestation, and encouraged them to protect forests [65,66]. At the same time, the stability of property rights also promotes farmers’ forestland transfer behavior, which increases the rural population in the non-farm sector. This eases rural dependence on forests for livelihoods and reduces the rate of deforestation. However, some scholars have denied the role of NRCFT on forest resource conservation, arguing that the short-sighted behavior of foresters may exacerbate deforestation [67,68]. Meanwhile, the planting of large areas of fast-growing forests by large forestry contractors in pursuit of economic interests [69], the crude management model exacerbated forest degradation [70].

Current approaches to measuring forest fragmentation are mainly from the fields of Cultural Geography and Landscape Ecology. The landscape index method [71,72,73] and the forest fragmentation model [74,75] are two mainstream approaches to measuring forest fragmentation. Both methods use remote sensing technology to obtain land cover classification products, then analyze them using software technologies such as ArcGIS and ENVI. The landscape index method belongs to the field of landscape ecology research. It evaluates the forest fragmentation by calculating the landscape pattern index [76,77,78,79]. The forest fragmentation model aggregates and classifies land use types into three categories: forested image elements, non-forested image elements, and missing image elements. Then uses a moving window algorithm to build a forest fragmentation model, which is used for describing forest fragmentation [80,81].

Forest fragmentation is influenced by local ecological and socio-economic development [82,83].

- Economic factors. Economic growth is considered to be the main driver of forest fragmentation [84,85]. Economic growth is accompanied by the input of land elements, which reveal the original value of land assets [86]. Meanwhile, the change of land function demanded by land operators causes land use transformation, leads to deforestation, and causes changes in forest spatial patterns [87].
- Policy factors. National policies determine the degree of natural resource protection [88]. Ecological construction projects implemented in China, such as the Three Norths Shelter Forest System Project, the Natural Forest Conservation Program, and the Grain for Green Program (GGP) have curbed the process of forest fragmentation [89]. Local governments, as the principal part of environmental governance, can prevent forest fragmentation by influencing the main behavior of land users and regulating regional land use patterns [90]. However, if local government departments are inefficient in management, poor interdepartmental coordination may lead to forest fragmentation [91].
- Demographic factors. Population increase is a potential driver of deforestation [92]. Rapid population growth can lead to increased demand for transportation, building, and farmland, which increases the exploitation of forestland [93,94], resulting in forest fragmentation. In addition to this, population migration during urbanization reduces the rural dependence on forests for livelihood. From another perspective, population migration causes a sharp decline in the rural labor force, which leads to the crude management of forest land and may trigger forest degradation.

In summary, NRCFT has gradually become the focus of academic attention. However, most of the studies on the impact of NRCFT on forest resources have focused on the perspective of forest area change [65,66], and few of them have conducted in-depth analysis from the perspective of forest fragmentation. The purpose of this paper is to take the Beijing–Tianjin–Hebei region in China as an example to explore the impact of NRCFT on forest fragmentation. This is an important direction for urgent research in the field of socio-economic drivers of forest fragmentation at present.

## 2. Data and Methods

### 2.1. Data Sources

**Figure 1:**
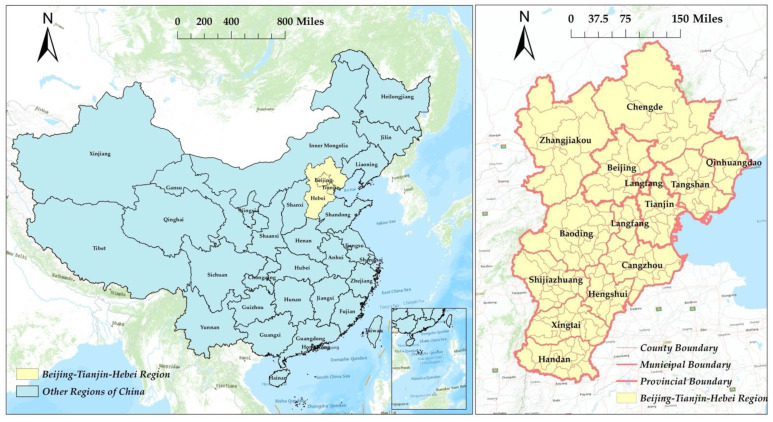
Location and range of the Beijing–Tianjin–Hebei region, China.

To explore the impact of NRCFT on forest fragmentation in the Beijing–Tianjin–Hebei region of China (Figure 1), 38 counties with high forest cover in the region were selected as the study sample. Taking into account the availability of data, Landsat 5 and Landsat 8 satellite remote sensing images (https://earthexplorer.usgs.gov/, accessed on 1 May 2021) in 2000, 2005, 2010, 2015, and 2018 were selected as data sources. The China Rural Statistical Yearbook and the China Statistical Yearbook (Township) of previous years to obtain socio-economic development data.

### 2.2. Forest Fragmentation Measurement Method

This study adopts the Landscape indices method. This method uses satellite remote sensing images as data, then uses GIS analysis technology for remote sensing interpretation, and finally uses Fragstats software (spatial pattern analysis software) to analyze and derive the Landscape indices. The process is as follows.

#### 2.2.1. Data Pre-Processing

Different reflectance for different wavelengths of sunlight or other forms of electromagnetic waves were exhibited by various land cover types. Table 1 outlines some of the phenomena that are revealed by each of the wavelength bands.

Landsat satellite remote sensing images were used to identify different feature characteristics by enhancing the images with pseudo-color synthesis technique, combined with the ultra-high spatial resolution images of Google Earth. Six types of features: woodland, grassland, agricultural land, bare land/building land, water bodies, and other sites were selected as training samples. Training samples were selected with the help of ultra-high spatial resolution images in Google Earth. For example, to select training samples for the forest class, draw polygons that are forests in Google Earth imagery and then locate these polygons on Landsat images to select forest samples. This method ensures that all samples are correctly selected. That is to say, there used ultra-high spatial resolution images to select samples.

#### 2.2.2. Remote Sensing Interpretation

1. Calculation of classification. Compute ROI Separability is used to calculate sample separability. After the samples are checked, the support vector machine (SVM) is used to classify Landsat images. SVM is a supervised classification algorithm that draws hyperplanes in n-dimensional space to differentiate samples. Before SVM classification, use the linear normalization to normalize the digital number in all spectral bands, which places the attribute numeric values on the same scale and prevents attributes of large original scales from biasing the solution. To use an SVM classifier, have to choose a kernel. which is a function that transforms the input data to a high-dimensional space, so that the data is separable and the problem can be solved in the new space. In ENVI, it has four types of kernels: linear, polynomial, RBF (Radial Basis Function), and sigmoid. This work chooses the linear kernel to perform the classification as it has higher efficiency than others. Then the image elements are judged one by one and output the classification result.
2. Correction of classification results. The training samples of the woodland are sampled by extracting the woodland part of each period. Then the second classification is performed by the neural network algorithm to obtain the corrected woodland distribution data.
3. Accuracy evaluation. Firstly, the validation points in the decoded area were randomly and uniformly selected to plot a confusion matrix, and the kappa coefficient was calculated to evaluate the classification accuracy [95]. Finally, the overall classification accuracy of images was calculated to be above 80%, and the kappa coefficient was above 0.7. Among them, the user accuracy of woodland ranged from 81.83% to 99.92%, indicating that 81.83% to 99.92% of the image elements classified as woodland were woodland, and the data availability was good.

#### 2.2.3. Calculation of Forest Fragmentation Indicators

The Landscape Shape Index (LSI) is usually used as a proxy variable for forest fragmentation indicators. In this study, Fragstats and Excel were used to calculate the landscape shape index. In addition, to ensure the rigor of the research results, Patch Density (PD) and Edge Density (ED) indices are also calculated for the robustness check later. The specific algorithms and their meanings are as follows:

- Landscape Shape Index (LSI)

(1) $$\mathrm{LSI}=\frac{0.25E}{\sqrt{A}}$$

where E is the total length of all patch boundaries in the landscape, A is the total area of the landscape. It reflects the complexity of shapes at different spatial scales. The range of values: LSI ≥ 1. When the shape of patches in the landscape is irregular or deviates from the square, LSI increases, indicating the increase in forest fragmentation;

- Patch Density (PD)

(2) $$\mathrm{PD}=\frac{N}{A}$$

where N is the total number of landscape patches, A is the total landscape area. It indicates the number of patches per square kilometer. It reflects the fragmentation degree of the landscape and the degree of spatial heterogeneity of the landscape. The value range: PD > 0. The higher the value of PD, the higher the degree of forest fragmentation;

- Edge Density (ED)

(3) $$\mathrm{ED}=\frac{E}{A}10^{6}$$

where E is the total length of all patch boundaries in the landscape, A is the total area of the landscape. It reflects the magnitude of the landscape that is being destroyed. The range of values: ED ≥ 0. The larger the value, the more fragmented the landscape pattern.

### 2.3. Variable Selection

Dependent Variables: The dependent variable is the degree of forest fragmentation. Based on the availability of data and drawing on existing studies, the Landscape Shape Index (LSI) is selected as a proxy variable to measure forest fragmentation. In addition, drawing from Liu et al. [83] and Su et al. [85], this paper uses Patch Density (PD) and Edge Density (ED) as proxy variables to conduct robustness tests on the baseline regression results.

Independent Variable: The core independent variable is the implementation of NRCFT, and the variable takes the value of 1 if the sample county has implemented NRCFT in the corresponding year, otherwise, it takes the value of 0.

Control Variables: Concerning existing literature, the following factors influencing forest fragmentation are added as control variables in this paper:

- Economic growth. The change in land use type brought about by economic growth has a significant effect on forest fragmentation. Based on existing studies and choose per capita disposable income as a proxy variable for economic growth [96];
- Grain for Green Program (GGP). GGP significantly increases the forest cover in China and helps to reduce forest fragmentation, so the implementation of GGP is added to the model as a control variable;
- Urbanization. From the land-use dimension, the urbanization process is the continuous transformation of large-scale forestry land into construction land [97], this causes a large reduction of forest area. Therefore, control for the effect of the urbanization rate on forest fragmentation;
- Rural energy consumption transformation. The transition of rural energy from fuelwood to electricity may significantly avoid deforestation behavior, thus reducing the probability of forest fragmentation. This paper uses rural per capita electricity consumption as a proxy variable for rural energy consumption transition;
- Intensive land use. The increase in intensive arable land use improves the output per unit of land and also relieves pressure on forests. Therefore, crop sown area per capita is added to the model as a proxy variable for the intensive land use to control its effect on forest fragmentation;
- Demographic factors. The increase in demand for food and fuel brought about by population growth leads to predatory deforestation. Therefore, in this paper, population density is used as a proxy variable for demographic factors.
- Transportation infrastructure construction. Transportation infrastructure construction may occupy or destroy forestland, and the road grid may exacerbate forest fragmentation. In this paper, the number of road miles per capita is used as a proxy variable for transportation infrastructure construction.

### 2.4. Model Construction

The regression model for this paper is constructed as follows:

(4) $${\mathrm{FF}}_{it}=\alpha_{it}+{\beta \mathrm{TREAT}}_{it}+\theta_{1}{\mathrm{INC}}_{it}+\theta_{2}{\mathrm{GGP}}_{it}+\theta_{3}{\mathrm{URBAN}}_{it}+\theta_{4}{\mathrm{ELE}}_{it}+\theta_{5}\mathrm{FAR}M_{it}\;+\theta_{6}{\mathrm{POPDEN}}_{it}+\theta_{7}{\mathrm{ROAD}}_{it}+\varepsilon_{it}\;$$

where i represents the sample county and t represents the year. ${\mathrm{FF}}_{it}$ is forest fragmentation, measured by the landscape shape index (LSI); ${\mathrm{TREAT}}_{it}$ is the proxy variable for NRCFT, ${\mathrm{TREAT}}_{it}=0$ if the county i does not implement NRCFT, ${\mathrm{TREAT}}_{it}=1$ if NRCFT has been implemented in county i; ${\mathrm{INC}}_{it}$ is disposable Per capita income; ${\mathrm{GGP}}_{it}$ is the dummy variable for the GGP, if the sample has implemented GGP in that year, the variable is assigned to 1, otherwise, it is assigned to 0; ${\mathrm{URBAN}}_{it}$ is the urbanization rate, which is a proxy variable for urbanization development; ${\mathrm{ELE}}_{it}$ is the rural electricity consumption per capita variable, which is a proxy variable for the transformation of rural energy consumption; $\mathrm{FAR}M_{it}$ is the crop sown area per capita variable, which is a proxy variable for the intensive use of arable land; ${\mathrm{POPDEN}}_{it}$ is the population density; ${\mathrm{ROAD}}_{it}$ is the road mileage per capita, which is a proxy variable for transportation infrastructure. Where β is the focus of attention in this study, capturing the effect of collective forest rights system reform on forest fragmentation; $\theta_{1}$–$\theta_{7}\;$ captures the effect of each control variable on forest fragmentation; $\varepsilon_{it}$ is the random error term.

## 3. Results

### 3.1. Descriptive Statistics of the Variables

As shown in Table 2, the mean value of the forest fragmentation index (LSI) decreased from 155.50 in 2000 and 157.99 in 2010 to 119.13 in 2018, indicating that the overall forest fragmentation in the Beijing–Tianjin–Hebei region showed a more significant decreasing trend during the period from 2010 to 2018. The per capita disposable income gradually increased from 1800 CNY in 2000 to 11,100 CNY in 2018, indicating that the level of economic development of the counties in the study area had a significant increase during this period, and the quality of people’s living standards had been significantly improved. The implementation ratio of GGP in the Beijing–Tianjin–Hebei region was 34% in 2000, while by 2010 the sample counties (districts) selected for this paper had all carried out the GGP. Significant growth in urbanization rate, which was 6.02% in 2000 and has reached 16.27% in 2018. From the proxy variables of rural energy transition, rural per capita electricity consumption gradually rose from 150.46 kWh/person in 2000 to 620.71 kWh/person in 2018. The crop sown area per capita has shown a decreasing trend in the past 20 years, from 1553.649 m^2^ in 2000, down to 1038.23 m^2^ in 2018. In terms of demographic factors, the population density showed a trend of rising and then falling between 2000 and 2018, over the time span of 2000 to 2018, it increased by 1.06 times. In terms of the proxy variable of transportation infrastructure, the road mileage per capita increased by nearly 232% between 2000 and 2018.

**Figure 2:**
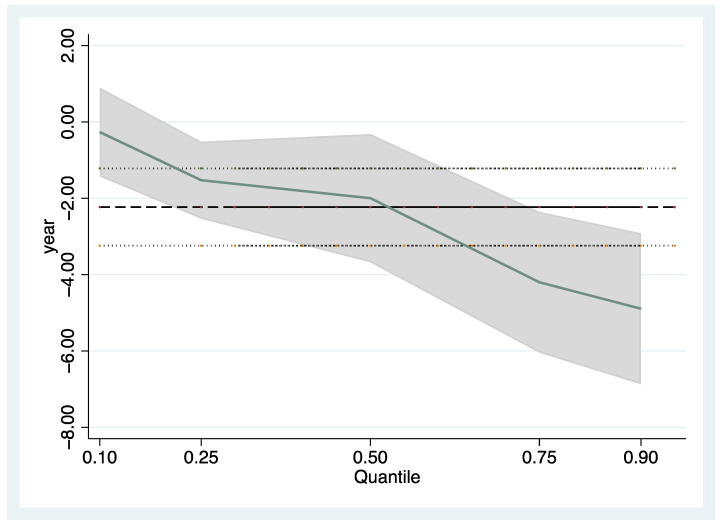
Change in panel quantile coefficients. Notes: *x*-axis represents the conditional quantile of (fragmentation), and the *y*-axis denotes the coefficient values of (year).

Figure 2 depicts the distinct patterns of change in forest fragmentation at different quantitative levels.

**Figure 3:**
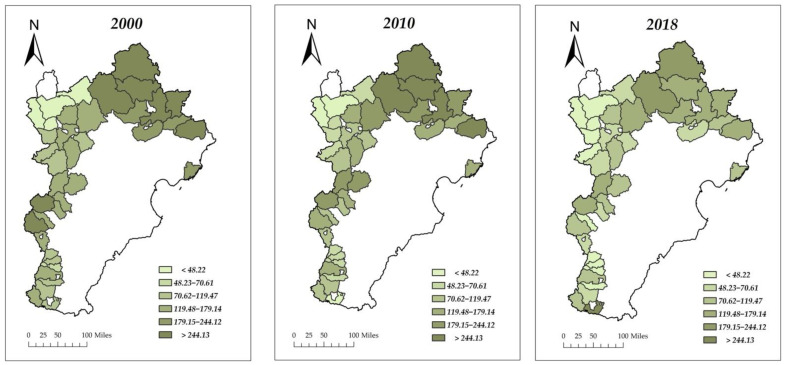
Changes in forest fragmentation in the Beijing–Tianjin–Hebei region, 2000, 2010, and 2018.

To represent the variation of forest fragmentation more visually, we plotted Figure 3. As shown in Figure 3, the darker the color, the higher the value of LSI, and the more fragmented the forest landscape pattern.

### 3.2. Empirical Results

Before the econometric regression, calculate the model variance inflation factor (VIF) to analyze whether there is a multicollinearity problem. When 0 < VIF < 10, it indicates that there is no multicollinearity. Table 3 shows that the maximum value of VIF is 2.460 and the minimum value is 1.190, which means there is no serious multicollinearity problem among the explanatory variables. Secondly, to choose between the fixed-effects model and the random-effects model, the Hausman test is conducted in this paper. The test results reject the original hypothesis of the random effect model, so this paper chooses the fixed-effect model for analysis.

The regression results are shown in Table 4. After gradually adding all control variables, NRCFT (TREAT) still has an effect on forest fragmentation level and is significant at the 5% confidence level, (i.e., NRCFT significantly inhibits forest fragmentation). Per capita, disposable income (INC) increases the forest fragmentation, and the second term of it (INC2) has a significant negative effect on forest fragmentation, indicating that per capita disposable income has an inverted U-shape relationship with forest fragmentation, i.e., at low-income levels, economic growth increases fragmentation, at high-income levels, economic growth leads to fragmentation improvement. Model 8 shows that after controlling for the effects of other factors, the implementation of the GGP significantly reduces forest fragmentation. Table 4 also indicates that rural per capita electricity consumption has a significant effect on forest fragmentation, the more electricity used in rural areas, the lower the level of forest fragmentation, suggesting that by realizing the transformation of rural energy consumption, fuelwood utilization and forest harvesting are effectively reduced, thus reducing forest fragmentation. Models 3–8 show that urbanization rate, per capita crop sown area, population density, and per capita road mileage have no significant effects on forest fragmentation after controlling for various factors.

### 3.3. Robustness Test

To further verify the robustness of the baseline model, this section performs robustness tests by replacing the forest fragmentation index LSI (landscape shape index) with PD (patch density index) and ED (edge density index), respectively.

In Table 5, the results of the core independent variable are consistent with the results of the benchmark regressions in Table 4, i.e., NRCFT significantly reduces forest fragmentation, which proves the robustness of the benchmark model findings. The coefficient of the primary term of INC is positive, and the coefficient of its quadratic term is negative and significant at the 5% confidence level, which proves the robustness of an inverted U-shape relationship between per capita disposable income and forest fragmentation. The significance of the GGP variable on forest fragmentation did not change, which verified the robustness of the results. Comparing model 8, model 11, and model 14, found that when the dependent variable was ED (marginal density index), the direction of the effect of rural per capita electricity consumption did not change but was no longer significant, which might be related to the sample measurement error. In addition, the significant effects of urbanization rate, crop sown area per capita, population density, and road mileage per capita on forest fragmentation did not change, which is consistent with the baseline regression results in Table 4. and proves the robustness of the results in this paper.

## 4. Discussion

### 4.1. Principal Findings

Firstly, NRCFT significantly reduces forest fragmentation in the Beijing–Tianjin–Hebei regions. Differ from the existing studies on the impact of NRCFT on forest cover or deforestation rate discussed in the literature [98,99], this study is concerned with spatial pattern changes of forests rather than area. Second, this paper verifies that forest fragmentation is an inverted U-shaped function of per capita disposable income, consistent with Li, L.C. [100]. This is important for exploring the relationship between economic growth and forest conservation. Thirdly, the implementation of GGP significantly reduces forest fragmentation, and the result is consistent with the analysis of Li, W. et al. [9]. The implementation of GGP in China was piloted in 1999 in Sichuan, Shaanxi, and Gansu provinces, and has been replicated on the full scale since 2002, as a reforestation activity implemented in China from the perspective of environmental protection. This study shows that GGP helps reduce forest fragmentation and optimize the spatial pattern of forests, which aims to increase forest cover. Fourth, rural energy transition significantly reduced forest fragmentation, extending the studies of Defries, R. et al. and Li, G.Z. et al. [101,102]. The shift in rural energy consumption structure from fuelwood and coal-based to electricity-based in the Beijing–Tianjin–Hebei region reduced fuelwood consumption thus leading to lower forest fragmentation. Finally, different from the existing literature that suggests that urbanization rate and transportation infrastructure construction may increase forest fragmentation [100,102], this study does not support this conclusion. The implementation of a strict land-use control system in China has curbed the effects of forest fragmentation due to urbanization and the construction of transportation infrastructure [103,104].

Regarding the reasons for which NRCFT has reduced forest fragmentation, the following reasons are possible: First, from the perspective of local governments, NRCFT has facilitated the use of indigenous knowledge by local governments. To effectively protect the forest and the integrity of spatial patterns by formulating forest management policies, which are more in line with local conditions. Second, from the market perspective, NRCFT has enabled local governments and the market to form a synergy of marketization of forest rights. Participation of market actors can overcome the inefficiency of government actions by injecting pressure into the supply of environmental services. Finally, from the community perspective, the stability of property rights has reduced the behavior of farmers carrying out large-scale logging. The government and market-driven mechanism of forest rights transfer encourage farmers to transfer forest land as an asset resource. The realization effect increases farmers’ investment in forestry and enhances forest resource management.

### 4.2. Strengths and Limitations

The existing literature provides a solid theoretical foundation for this study and provides a methodological guide for the empirical analysis. The innovation of this paper is to verify the impact of NRCFT on forest resources from an empirical perspective, which enriches the research on forest fragmentation from a social disciplinary perspective.

The shortcoming is the lack of an analysis of the impact mechanisms of NRCFT on forest fragmentation from an empirical perspective, which is an important direction for future research.

### 4.3. Implications

This paper verifies that NRCFT helps reduce forest fragmentation, but there are still many problems in the implementation of NRCFT. Such as disputes over mountains, forest ownership, and forestry taxes issues. So, the government must accelerate the implementation of supporting policies for NRCFT to maintain the protective effect of NRCFT on forest resources. Synergistic economic growth and ecological development are achievable, a high-quality economic growth model should be explored. Given the importance of the GGP to forest resources, more attention should be paid to the follow-up support of this policy. In addition, implementing the energy revolution in rural areas may be an effective way to conserve forest resources.

## 5. Conclusions

This study reveals the relationship between NRCFT and forest fragmentation from the perspective of spatial distribution. Although some limitations exist, it enriches the study of forest fragmentation from a social disciplinary perspective. The results suggest that NRCFT helps reduce forest landscape fragmentation, and it is vital to focus on the implementation of GGP, high-quality economic development, and rural energy transition.

## Figures and Tables

**Table 1:** Phenomena revealed by different bands of Landsat data.

Band	Phenomena Revealed
0.45–0.52 µm (visible blue)	Identify water bodies, soil, and vegetation
0.52–0.60 µm (visible green)	Measure the peak reflection of green light from vegetation
0.63–0.69 µm (visible red)	Detect chlorophyll absorption and identify vegetation types
0.76–0.90 µm (near IR ^1^)	Identify vegetation type and biomass, as well as water and soil moisture
1.55–1.75 µm (mid IR)	Identify the water content of soil and vegetation
10.40–12.50 µm (thermal IR)	Identify the degree of plant stress, soil moisture, and to measure surface heat
2.08–2.35 µm (mid IR)	Distinguish mineral and rock types

^1^ IR means Infrared Region.

**Table 2:** Descriptive statistics of the variables.

Variables	Indicators	2000	2010	2018
		Mean (st.)	Mean (st.)	Mean (st.)
LSI	Forest fragmentation	155.50 (84.19)	157.99 (85.90)	119.13 (59.17)
INC	Per capita disposable income (10^4^ CNY)	0.18 (0.07)	0.43 (0.15)	1.11 (0.26)
GGP	Implementation of GGP (%)	0.342 (0.48)	1 (0.00)	1 (0.00)
URBAN	Urbanization rate	6.02 (6.54)	13.38 (3.73)	16.27 (9.46)
ELE	Rural electricity (kWh/per)	150.4 (108.49)	558.41 (681.54)	620.71 (709.58)
FARM	Crop sown area (m^2^)	1553.649 (1173.84)	1189.87 (627.20)	1038.23 (798.36)
POPDEN	Population density (people/ha)	186.12 (137.07)	200.74 (143.99)	198.21 (138.78)
ROAD	Road mileage (m/per)	2.24 (1.42)	4.37 (1.778)	5.2 (1.86)

Standard errors in parentheses.

**Table 3:** Results of multicollinearity diagnostic.

Variables	VIF	1/VIF
TREAT	2.460	0.407
INC	2.350	0.425
ROAD	2.230	0.448
POPDEN	1.910	0.522
GGP	1.400	0.713
URBANI	1.390	0.719
FARM	1.270	0.789
ELE	1.190	0.838

**Table 4:** Regression results of the benchmark model.

Variables	Model 1	Model 2	Model 3	Model 4	Model 5	Model 6	Model 7	Model 8
	LSI	LSI	LSI	LSI	LSI	LSI	LSI	LSI
TREAT	−0.230 ***	−0.711 ***	−0.419 *	−0.448 **	−0.489 **	−0.497 **	−0.541 **	−0.546 **
	(−0.063)	(−0.242)	(−0.221)	(−0.219)	(−0.221)	(−0.22)	(−0.221)	(−0.221)
INC		1.058 **	1.058 **	0.927 *	1.078 **	1.110 **	1.194 **	1.223 **
		(−0.511)	(−0.511)	(−0.491)	(−0.521)	(−0.524)	(−0.543)	(−0.546)
INC2		−0.399 **	−0.399 **	−0.325 *	−0.381 **	−0.395 **	−0.425 **	−0.430 **
		(−0.176)	(−0.176)	(−0.163)	(−0.177)	(−0.180)	(−0.188)	(−0.19)
GGP			−0.292 ***	−0.324 ***	−0.318 ***	−0.336 ***	−0.344 ***	−0.333 ***
			(−0.053)	(−0.060)	(−0.060)	(−0.060)	(−0.069)	(−0.072)
URBAN				0.894 ***	0.859 ***	0.675 *	0.674	0.72
				(−0.323)	(−0.308)	(−0.400)	(−0.403)	(−0.437)
ELE					−0.692 **	−0.655 *	−0.672 *	−0.665 *
					(−0.340)	(−0.328)	(−0.333)	(−0.330)
FARM						−0.678	−0.657	−0.675
						(−0.943)	(−0.943)	(−0.964)
POPDEN							0.604	0.446
							(−1.252)	(−1.332)
ROAD								−4.985
								(−7.459)
Constant	4.865 ***	4.693 ***	4.693 ***	4.660 ***	4.648 ***	4.758 ***	4.630 ***	4.677 ***
	(−0.032)	(−0.101)	(−0.101)	(−0.092)	(−0.093)	(−0.171)	(−0.307)	(−0.328)
Observations	190	190	190	190	190	190	190	190
Number of id	38	38	38	38	38	38	38	38
R-squared	0.370	0.385	0.385	0.407	0.415	0.417	0.418	0.418

Standard errors in parentheses. *** p<0.01, ** p<0.05, * p<0.10.

**Table 5:** Robustness test.

Variables	Model 9	Model 10	Model 11	Model 12	Model 13	Model 14
	PD	PD	PD	ED	ED	ED
TREAT	−0.720 ***	−0.991 **	−0.757 *	−12.370 ***	−37.150 **	−25.310 **
	(−0.097)	(−0.374)	(−0.388)	(−3.799)	(−14.740)	(−12.270)
INC		1.221	1.482		54.780 *	63.280 **
		(−0.813)	(−0.935)		(−28.130)	(−26.430)
INC2		−0.672 **	−0.721 **		−20.960 **	−22.300 **
		(−0.328)	(−0.35)		(−9.873)	(−9.348)
GGP			−0.412 ***			−24.460 ***
			(−0.138)			(−5.184)
URBAN			1.202			44.530
			(−1.244)			(−28.080)
ELE			−1.574 **			−8.193
			(−0.737)			(−21.430)
FARM			−0.351			−43.980
			(−2.020)			(−34.790)
POPDEN			−0.194			72.610
			(−2.688)			(−60.110)
ROAD			−10.750			−243.200
			(−15.240)			(−709.500)
Constant	1.696 ***	1.502 ***	1.547 **	65.630 ***	56.940 ***	47.470 ***
	(−0.0476)	(−0.154)	(−0.69)	(−2.484)	(−5.662)	(−12.610)
Observations	190	188	188	190	188	188
Number of id	38	38	38	38	38	38
R-squared	0.394	0.405	0.427	0.389	0.406	0.449

Standard errors in parentheses. *** p<0.01, ** p<0.05, * p<0.10.

## Data Availability

The data presented in this study are available on request from the corresponding author.

## Abbreviations

NRTFP	New wave of Reform of Collective Forestland Tenure
GGP	Grain for Green
IR	Infrared Region
LSI	Landscape Shape Index
PD	Patch Density
ED	Edge Density
VIF	Variance Inflation Factor
